# Host transcriptional responses following *ex vivo* re-challenge with *Mycobacterium tuberculosis* vary with disease status

**DOI:** 10.1371/journal.pone.0185640

**Published:** 2017-10-04

**Authors:** Elaine A. Yu, Serene H. John, Elizabeth C. Tablante, Christine A. King, John Kenneth, David G. Russell, Saurabh Mehta

**Affiliations:** 1 Division of Nutritional Sciences, Cornell University, Ithaca, New York, United States; 2 Division of Infectious Diseases, St. John’s Research Institute, Bangalore, Karnataka, India; 3 Department of Microbiology and Immunology, State University of New York Upstate Medical University, Syracuse, New York, United States; 4 Department of Microbiology and Immunology, College of Veterinary Medicine, Cornell University, Ithaca, New York, United States; 5 Institute for Nutritional Sciences, Global Health, and Technology, Cornell University, Ithaca, New York, United States; Institut de Pharmacologie et de Biologie Structurale, FRANCE

## Abstract

The identification of immune correlates that are predictive of disease outcome for tuberculosis remains an ongoing challenge. To address this issue, we evaluated gene expression profiles from peripheral blood mononuclear cells following *ex vivo* challenge with *Mycobacterium tuberculosis*, among participants with active TB disease (ATBD, n = 10), latent TB infection (LTBI, n = 10), and previous active TB disease (after successful treatment; PTBD, n = 10), relative to controls (n = 10). Differential gene expression profiles were assessed by suppression-subtractive hybridization, dot blot, real-time polymerase chain reaction, and the comparative cycle threshold methods. Comparing ATBD to control samples, greater fold-increases of gene expression were observed for a number of chemotactic factors (CXCL1, CXCL3, IL8, MCP1, MIP1α). ATBD was also associated with higher IL1B gene expression, relative to controls. Among LTBI samples, gene expression of several chemotactic factors (CXCL2, CXCL3, IL8) was similarly elevated, compared to individuals with PTBD. Our results demonstrated that samples from participants with ATBD and LTBI have distinct gene expression profiles in response to *ex vivo M*. *tuberculosis* infection. These findings indicate the value in further characterizing the peripheral responses to *M*. *tuberculosis* challenge as a route to defining immune correlates of disease status or outcome.

## Introduction

Globally, 10.4 million incident cases of active tuberculosis (TB) disease (ATBD) and 1.4 million TB-related deaths were reported in 2015 [1]. Over the past century, anti-TB drugs, bacille Calmette-Guérin (BCG) vaccination, and public health strategies such as directly observed treatment, have contributed to a reduction in TB-related mortality [1]. However, latent TB infection (LTBI) and recurrent ATBD remain critical issues for global control [1, 2], particularly given that previous anti-TB treatment is an established risk factor for drug resistance [3, 4].

Determining the contribution of *Mycobacterium tuberculosis* re-infection, as opposed to reactivation, is a challenge for numerous TB control programs, especially in endemic areas [5]. *M*. *tuberculosis* re-infection can occur at any time during ATBD and LTBI, and is independent of relapse [5]. Moreover, the inability of vaccination to protect against *M*. *tuberculosis* re-infection and reactivation represent significant gaps in research and therapeutics [6–8]. The sole approved vaccine BCG and vaccines in the development pipeline are largely protective against ATBD [9], and not LTBI or exogenous *M*. *tuberculosis* re-infection [8, 10]. Currently there is no approved and effective vaccine for individuals with LTBI [8, 10].

The assessment of vaccine efficacy hinges on identifying biomarkers predictive of disease progression and outcomes [8, 11, 12]. Given the numerous constraints for TB vaccine development, it has been hypothesized that identification of peripheral correlates of protective immunity against *M*. *tuberculosis* may be more realistic, compared to a single biomarker [13–15]. A unique transcriptional signature has been identified for ATBD; this whole-blood transcript signature was associated with disease severity and observed to resolve after treatment [16]. The extent to which this signature is predictive rather than diagnostic still needs to be determined. Previous studies have argued that differential gene expression was associated with TB disease recurrence, susceptibility, and host control [17–19]. Little is known regarding transcriptional biomarkers of post-primary *M*. *tuberculosis* re-infection or even enhanced exposure due to reactivation, despite the public health significance.

Distinguishing gene expression patterns following *ex vivo* challenge with *M*. *tuberculosis* among individuals with ATBD, LTBI, previous active TB disease (PTBD; after successful treatment) has significance in identifying diagnostic and predictive biomarkers, which are required for development of vaccines and therapeutics [20, 21]. Our study objective involved delineating patient responses to the *ex vivo* challenge of *M*. *tuberculosis* through analysis of the relative gene expression profiles between study participants with ATBD, LTBI, PTBD, compared to controls.

## Materials and methods

### Ethical conduct of research

The Institutional Ethics Review Committees at St. John’s Medical College and Hospital (St. John’s National Academy of Health Sciences; Bangalore, Karnataka, India) and Arogyavaram Medical Centre (Arogyavaram, Andhra Pradesh, India) approved the study protocol. Study participants provided voluntary informed consent prior to data collection.

### Study population

Study participants (n = 40) were enrolled at a hospital outpatient department (St. John’s Medical College and Hospital, Bangalore, Karnataka) in India. Participants included four groups of patients with ATBD (n = 10) and LTBI (n = 10), PTBD (n = 10), and controls (n = 10). Inclusion criteria included TB status (defined below), and BCG vaccination. Exclusion criteria included: age (≤ 14 years), HIV infection (GS HIV Combo Ag/Ab EIA; Bio-Rad Laboratories, Redmond, Washington, United States).

Definitions of TB status included: ATBD, LTBI, and PTBD. ATBD status was based on acid-fast bacilli (AFB) sputum smear microscopy, which was performed by standard protocol (Ziehl-Neelsen staining), or Xpert MTB/RIF (Cepheid, Sunnyvale, California, United States). Patients with ATBD were newly diagnosed and had not initiated anti-TB treatment (or received <1 week of treatment). LTBI was diagnosed by QuantiFERON-TB Gold In-Tube (QFT-G; Cellestis Limited, Carnegie, Victoria, Australia). Blood samples were collected in QFT-G tubes, assayed according to manufacturer protocol and by enzyme-linked immunosorbent assay. Study enrollment was based on a positive QFT-G diagnostic result. PTBD was defined as having previous ATBD (pulmonary), anti-TB treatment, and post-treatment sputum conversion. PTBD patients received 6 months of anti-TB treatment for ATBD; study enrollment occurred between 2–371 days after completing treatment. Controls were considered individuals with a negative QFT-G diagnostic result.

### Sample collection and peripheral blood mononuclear cell isolation

Venous blood samples (8 mL) were collected in mononuclear cell preparation tubes (CPT^™^; Becton Dickinson Vacutainer Systems; Franklin Lakes, New Jersey, United States), and processed per manufacturer’s instructions.

Peripheral blood mononuclear cells (PBMCs) were isolated from blood samples, based on manufacturer instructions (CPT^™^; Becton Dickinson Vacutainer Systems; Franklin Lakes, New Jersey, United States). PBMCs were suspended at 5 x 10^6^ cells/mL in cryopreservation medium (45% RPMI 1640, 45% fetal bovine serum, 10% dimethyl sulfoxide), and incubated overnight at -80°C (in Mr. Frosty^™^ freezing container; Nalgene, Rochester, New York, United States). PBMCs were stored in liquid nitrogen prior to analyses.

### *Mycobacterium tuberculosis* infection

Frozen PBMCs were thawed and incubated overnight (37°C in a 5% CO_2_ humidified incubator) in growth media (RPMI 1640 [Gibco Laboratories; Grand Island, NY] with 2mM glutamine, 10% fetal bovine serum, 10mM 2-[4-[2-hydroxyethyl]-1-piperazinyl] ethanesulfonic acid, [Antibiotic-Antimycotic solution; Gibco Laboratories; Grand Island, New York, United States]). Cells (10^6^) were washed and re-suspended in fresh growth media containing 2 μg/ml whole cell lysate of *M*. *tuberculosis* strain H37Rv (*Mycobacteria* Research Laboratories at Colorado State University, Colorado, United States) for 48 hours (37°C in a 5% CO_2_ humidified incubator). An initial pilot study standardized the lysate concentration required for stimulation.

### Ribonucleic acid isolation

Total ribonucleic acid (RNA) was extracted (RNA Easy Plus Kit; Qiagen, Hilden, Germany) and quantitated (Qubit^®^ 2.0 fluorometer; Life Technologies, Milan, Italy) for suppression subtractive hybridization (SSH). In two patient groups (ATBD, LTBI), 300 ng total RNA from each individual were pooled and quantified. Pooled total RNA (300 ng) was used for complementary deoxyribonucleic acid (cDNA) synthesis (SMARTer^™^ polymerase chain reaction [PCR] cDNA Synthesis Kit; Clontech Laboratories, Inc., Palo Alto, California, United States).

### Complementary deoxyribonucleic acid subtractive libraries

SSH libraries were created with the PCR-select cDNA subtraction kit (Clontech Laboratories, Inc.; Palo Alto, California, United States), per the manufacturer instructions. In forward subtraction (ATBD-LTBI), cDNA from the ATBD group was the tester and LTBI was the driver. Conversely, in reverse subtraction (LTBI-ATBD), LTBI cDNA was the tester and ATBD cDNA was the driver. Real-time PCR (Rotor Gene 6000; Qiagen Inc., Hilden, Germany) amplified the subtracted, unsubtracted, and control cDNA with M13 primers. Following PCR (DNA amplification), products were cloned in the PCR 2.1-TA vector (Invitrogen, Carlsbad, California, United States) and transformed into *Escherichia coli* Top10 cells. From forward subtraction, approximately 200 clones were obtained; in reverse subtraction, about 150 clones were obtained. Subtraction efficiency was evaluated by comparing glyceraldehyde-3-phosphate dehydrogenase expression in the subtracted and unsubtracted cDNA.

### Dot blot hybridization

PCR product (5 μl) were transferred to two nylon membranes. The two identical membranes were hybridized with a tester and driver probe. Digoxigenin (DIG)-labeled probes (DIG-High Prime Labelling and Detection kit; Roche Diagnostics; Mannheim, Germany) visualized hybridization by a color reaction. The detected (differentially expressed) clones were sequenced (ABI 3730 DNA Analyzer; Applied Biosystems, Foster City, California, United States). Sequences were compared with the National Center for Biotechnology Information (NCBI; National Institutes of Health) reference database (Basic Local Alignment Search Tool [BLAST]).

### PCR quantification of gene expression

Gene expression of the identified sequences (from hybridization) were further assessed with real-time PCR (Rotor Gene 6000; Qiagen Inc., Hilden, Germany) in replicate. Primers were designed with NCBI Primer-BLAST (S1 Table). Each PCR reaction included: 0.3 μg cDNA, 200 nM primers, 12.5 μL 2x KAPA SYBR FAST quantitative polymerase chain reaction (qPCR; KAPA Biosystems; Boston, Massachusetts, United States). PCR cycling conditions were: 1 cycle (95°C for 3 minutes); 35 cycles (95°C for 30 seconds, 60°C for 1 minute). Human acidic ribosomal protein (HUPO) was the internal control gene. Cycle thresholds (C_T_) were calculated through the Rotor Gene software (version 1.7.87).

### Statistical analysis

Relative gene expression was reported as fold-change, based on the comparative cycle threshold (2^-ΔΔCT^) method [22] with HUPO as the internal control gene. Specifically:

$${\text{Fold-change}\;=\;2}^{-}{}^{\Delta \Delta \text{CT}},$$ (1)

where

$$2^{-\Delta \Delta \text{CT}}=2^{-\left(\left(\left[\text{CT}\;\text{target}\;\text{gene-CT}\;\text{HUPO}\right]\;\;\text{sample}\;\text{A}\right)-\left(\left[\text{CT}\;\text{target}\;\text{gene}\;\text{-}\;\text{CT}\;\text{HUPO}\right]\;\;\text{sample}\;\text{B}\right)\right)}$$ (2)

[22]

For each comparison, fold-change was reported as the average of the fold-changes of the two replicates (Table 1).

**Table 1:** Pairwise comparison groups^a^.

ATBD	Controls
LTBI	Controls
PTBD	Controls
ATBD	LTBI
ATBD	PTBD
LTBI	PTBD

^a^Abbreviations: active TB disease (ATBD), latent TB infection (LTBI), previous active TB disease (PTBD; after successful treatment)

For sociodemographic characteristics, the normality assumption of the age variable was assessed by the Kolmogorov-Smirnov test. Comparisons between the four study participant groups were evaluated by Kruskal-Wallis and Fisher’s exact tests. Statistical analysis was conducted with SAS (version 9.4; SAS Institute, Inc., Cary, North Carolina, United States); statistical significance was based on alpha value < 0.05 and two-tailed tests.

## Results

### Study population

Among the study participants, 65.0% were male (Table 2). The proportion of men differed across TB status (ATBD, LTBI, PTBD, controls) (p = 0.03). The median age was 31.5 years (interquartile range [IQR] 27.0–40.5; Table 2), and ranged between 18–65 years. Median age was similar in the four study participant groups (p = 0.32).

**Table 2:** Study participant characteristics (n = 40)^a^.

**Males**, n (%)	26 (65.0)
**Age** (years), median (IQR)	31.5 (27.0, 40.5)
**TB**, n (%)	
ATBD ^b^	10 (25.0)
LTBI ^c^	10 (25.0)
PTBD ^d^	10 (25.0)
No TB (control) ^e^	10 (25.0)

^a^Abbreviations: active TB disease (ATBD), latent TB infection (LTBI), previous active TB disease (PTBD; after successful treatment).

^b^Acid-fast bacilli [AFB] sputum smear microscopy

^c^Individuals with a positive QuantiFERON Gold In-tube and negative AFB diagnostic

^d^Individuals with previous ATBD who recently received anti-TB treatment for pulmonary TB (2–371 days prior to study enrollment), and post-treatment sputum conversion (based on AFB)

^e^Controls were considered individuals with negative QFT-G and AFB diagnostic results.

### Active TB disease associated with increased cytokine gene expression

**Fig 1:**
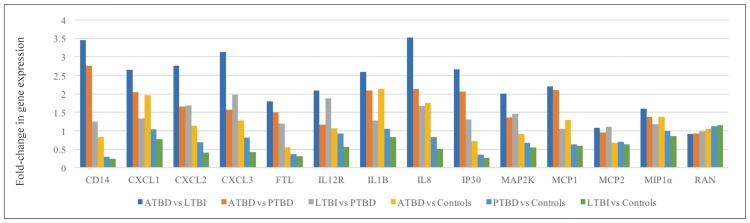
Relative gene expression among patients with ATBD, LTBI, PTBD, and controls^a^. ^a^ Abbreviations: active TB disease (ATBD), chemokine (C-X-C motif) ligand: (CXCL), cluster of differentiation (CD), ferritin light chain (FTL), gamma-interferon-inducible protein (IP30), interleukin (IL), latent TB infection (LTBI), mitogen activated protein kinase kinase (MAP2K), macrophage inflammatory protein (MIP), monocyte chemotactic protein (MCP), previous active TB disease (PTBD; after successful treatment), Ras-related nuclear protein (RAN).

After *M*. *tuberculosis* infection, ATBD patient samples had increased gene expression of chemotactic factors (chemokine [C-X-C motif] ligand 1 [CXCL1], 3 [CXCL3], 8 [CXCL8 or interleukin [IL]-8]; chemokine [C-C motif] ligand 2 [CCL2; monocyte chemotactic protein [MCP]1; macrophage inflammatory protein [MIP] 1α] compared to controls (Fig 1, S2 Table). The fold-increases in chemokine expression were also greater among ATBD samples (2.7-fold CXCL1, 2.8-fold CXCL2, 3.1-fold CXCL3, 3.5-fold CXCL8, 2.2-fold CCL2, 1.6-fold MIP1α), compared to LTBI (Fig 1, S3 Table). In contrast, CCL8 (MCP2) gene expression was similar in comparing ATBD versus LTBI 1.1-fold (S3 Table).

Relative to controls, ATBD was associated with higher gene expression of ILs (2.1-fold IL1B; Fig 1; S2 Table), which regulate the T helper 2 (Th2) response. In comparison to LTBI, ATBD samples had elevated IL1B (2.6-fold) and IL-12R (2.1-fold) gene expression (Fig 1).

### Latent TB infection versus previous active TB disease

In comparing LTBI against PTBD groups, several chemotactic factors were elevated (1.7-fold CXCL2, 2.0-fold CXCL3, 1.7-fold CXCL8 [IL-8]) although others were similar (1.1-fold CCL2 [MCP1]; Fig 1).

## Discussion

In summary, the gene expression profiles in ATBD, LTBI, PTBD, and control human study participants exhibit distinct patterns. These data provide a foundation for characterizing biomarker panels as correlates of protective immunity, which would serve as valuable surrogates in future development of TB vaccines and therapeutics.

Our findings are consistent with previous literature that observed elevated innate immune responses to *M*. *tuberculosis*, as well as *M*. *tuberculosis* evasion tactics in ATBD [23–28]. Similar to our results, other studies have established the importance of cytokines (including constitutive and stimulated chemokine gene expression such as MCP-1, IL-8, MIP1α) in host protection against *M*. *tuberculosis* [24–28]. *M*. *tuberculosis* has been shown to induce IL1B in dendritic cells, which upregulates the host Th2 response and dampens the protective Th1 response [23].

Additionally, similar to our results other data indicated that the gene expression of chemokines (including MIP1α) may differ during ATBD. Studies have identified a role for microbial lipoproteins in stimulating cytokine production (such as IL-12) in macrophages (via Toll-like receptors) [29]. MIP has also been found to be present in infectious pathogens, such as *Chlamydial trachomatis* [30].

Broadly, other studies have also highlighted the potential of biomarker panels (including host protein biosignatures) as indicators of the immune response against *M*. *tuberculosis* that could have diagnostic and/or predictive value [14–16, 31]. Furthermore, prior studies contend that differential gene expression was associated with increased risk of TB disease progression and relapse [17–19]. One study with whole-blood microarray analysis reported differential gene expression between patients with ATBD and LTBI, and identified gene expression profiles associated with host control of *M*. *tuberculosis* (specifically apoptosis and natural killer cell activity) [17]. Two studies indicated that differential gene expression profiles could associate with TB relapse [18, 19]. These studies show that biomarker profiles have potential to be more robust than single biomarkers of TB immunity. Interferon (IFN)-gamma, for example, is perhaps the most utilized candidate TB biomarker for a protective immune response against *M*. *tuberculosis* [32–35]; however IFN-gamma has low accuracy and predictive power, especially as a biomarker of protection or disease outcome [33].

Our study has several limitations. Firstly, PBMCs were cryopreserved prior to *M*. *tuberculosis* stimulation and RNA extraction, which could affect transcriptional processes. Secondly, the interpretation of gene expression in biological function remains preliminary [36]. Additionally, given the use of 2^-ΔΔCT^ as a relative gene expression quantitation method [22], the findings need to be confirmed and extended with additional housekeeping controls [37]. Due to the pooling of samples in each group, there was no variance data, which restricted statistical analyses and corrections for multiple hypothesis testing. Further confirmatory studies could alternatively utilize groupwise comparisons.

Future studies are needed to elucidate the role of transcriptional signatures as immune correlates (including via single cell analysis [38, 39], prior to any claims that such profiles are predictive of clinical morbidities (ATBD, LTBI) and treatment outcomes. Studies to date have assessed cross-sectional data and therefore have not allowed for causal inference, which we could potentially address in a human cohort. Furthermore, studies need to account for other potential confounding factors, including the heterogeneity of patient immune response [40, 41]. Nonetheless, these data provide a useful platform in defining initial immunological themes that allow us to differentiate between human patients that fall into the active (ATBD), latent (LTBI), and active-treated (PTBD) disease classes.

## Supporting information

S1 Table Primers in real-time PCR of differentially expressed genes. (DOCX)

S2 Table Relative gene expression among patients with ATBD, LTBI, PTBD, compared to controls. (DOCX)

S3 Table Relative gene expression among patients with ATBD, compared to LTBI. (DOCX)
