# Supplementary material for: Host transcriptional responses following ex vivo re-challenge with Mycobacterium tuberculosis vary with disease status
Source: PLoS One. 2017 Oct 4;12(10):e0185640. doi: 10.1371/journal.pone.0185640 (PMC5627917; doi:10.1371/journal.pone.0185640)
Supplement: S1 Table — (DOCX) [file pone.0185640.s001.docx]

**S1 Table: Primers in real-time PCR of differentially expressed genes ^a^**

| **Gene and Primer Name** | **Accession Number** | **Primer** | **Sequence** | **Tm**  (°C) | **Amplicon Size**  (bp) |
| --- | --- | --- | --- | --- | --- |
| CD14 | NM_001174105.1 | Forward | TTGCCCAAGCACACTCGCCT | 65.1 | 142 |
|  |  | Reverse | ATGGCCGGGAACTTGTGGGG | 65.0 |  |
| CXCL1 | NM_001511.3 | Forward | GCTTGCCTCAATCCTGCATCCC | 64.1 | 171 |
|  |  | Reverse | GGCCTCTGCAGCTGTGTCTCT | 64.4 |  |
| CXCL2 | NM_002982.3 | Forward | AGCTTGTCTCAACCCCGCATCG | 65.4 | 189 |
|  |  | Reverse | TTAGGCGCAATCCAGGTGGC | 63.2 |  |
| CXCL3 | NM_002090.2 | Forward | ACTGCGCCCAAACCGAAGTCA | 65.1 | 177 |
|  |  | Reverse | ACCACCCTGCAGGAAGTGTCAA | 64.1 |  |
| FTL | BC013928.1 | Forward | AGAAGCGCGAGGGCTACGAG | 64.4 | 189 |
|  |  | Reverse | GGGCAGAACCCAGGGCATGAA | 65.1 |  |
| IL12R | NM_001290024.1 | Forward | TCCTGCCCGTGTAAGGCCAA | 65.2 | 165 |
|  |  | Reverse | AGCCACTGGTTCTGTGTGGG | 63.6 |  |
| IL1B | BC008678.1 | Forward | CTGGTTCCCTGCCCACAGACC | 65.0 | 160 |
|  |  | Reverse | GCTGTGAGTCCCGGAGCGTG | 65.0 |  |
| IL8 | NM_000584.3 | Forward | CTGCAGCTCTGTGTGAAGGTGCAGT | 67.34 | 147 |
|  |  | Reverse | CTGTGTTGGCGCAGTGTGGTCC | 66.56 |  |
| IFI30 | NM_006332.3 | Forward | GTGCCCTACGGAAACGCACAG | 65.0 | 200 |
|  |  | Reverse | AGCTGCAGGCATAGTGGCAGA | 65.2 |  |
| MAP2K | NM_002755.3 | Forward | GGAGGAAGCGAGAGGTGCTGC | 65.4 | 159 |
|  |  | Reverse | CAAGGCCTCCAAGTTGGTCTCCG | 65.4 |  |
| MCP1 | NM_002982.3 | Forward | AAGCTCGCACTCTCGCCTCC | 64.7 | 128 |
|  |  | Reverse | ACAGCAGGTGACTGGGGCATT | 64.4 |  |
| MCP2 | NM_005623.2 | Forward | ACGGGGCAAGGAGGTCTGTGCTG | 68.86 | 167 |
|  |  | Reverse | TGCACCTGGGGGAGGTTGGGGA | 69.76 |  |
| MIP1α | NM_002983.2 | Forward | GGGCAGCAGACAGTGGTCAG | 63.1 | 164 |
|  |  | Reverse | GGCGTGTCAGCAGCAAGTGA | 63.6 |  |
| RAN | BC016654.1 | Forward | ACGCTTCTGGAAGGAACGCC | 64.0 | 165 |
|  |  | Reverse | GAACCTCAACACCCAAGGTGGC | 64.9 |  |

^a^ Abbreviations: active TB disease (ATBD), chemokine (C-X-C motif) ligand: (CXCL), cluster of differentiation (CD), ferritin light chain (FTL), gamma-interferon-inducible protein (IP30), interleukin (IL), latent TB infection (LTBI), melting temperature (Tm), mitogen activated protein kinase kinase (MAP2K), macrophage inflammatory protein (MIP), monocyte chemotactic protein (MCP), polymerase chain reaction (PCR), previous active TB disease (PTBD; after successful treatment), Ras-related nuclear protein (RAN)
