# Supplementary material for: Host transcriptional responses following ex vivo re-challenge with Mycobacterium tuberculosis vary with disease status
Source: PLoS One. 2017 Oct 4;12(10):e0185640. doi: 10.1371/journal.pone.0185640 (PMC5627917; doi:10.1371/journal.pone.0185640)
Supplement: S2 Table — (DOCX) [file pone.0185640.s002.docx]

**S2 Table: Relative gene expression among patients with ATBD, LTBI, PTBD, compared to controls** ^a^

|  | **2^-∆∆CT^** | | | | | | | | | | | | | |
| --- | --- | --- | --- | --- | --- | --- | --- | --- | --- | --- | --- | --- | --- | --- |
|  | CD14 | CXCL1 | CXCL2 | CXCL3 | FTL | IL12R | IL1B | IL8 | IP30 | MAP2K | MCP1 | MCP2 | MIP1α | RAN |
| ATBD ^b^ | 0.8 | 1.9 | 1.1 | 1.3 | 0.5 | 1.1 | 2.1 | 1.8 | 0.7 | 0.9 | 1.3 | 0.7 | 1.4 | 1.1 |
| LTBI ^c^ | 0.2 | 0.8 | 0.4 | 0.4 | 0.3 | 0.6 | 0.8 | 0.5 | 0.3 | 0.5 | 0.6 | 0.6 | 0.9 | 1.1 |
| PTBD ^d^ | 0.3 | 1.0 | 0.7 | 0.8 | 0.4 | 0.9 | 1.1 | 0.8 | 0.3 | 0.7 | 0.6 | 0.7 | 1.0 | 1.1 |
| No TB (control) ^e^ | --- | --- | --- | --- | --- | --- | --- | --- | --- | --- | --- | --- | --- | --- |

^a^ Abbreviations: acid-fast bacilli (AFB), active TB disease (ATBD), chemokine (C-X-C motif) ligand: (CXCL), cluster of differentiation (CD), cycle threshold (C_T_), ferritin light chain (FTL), gamma-interferon-inducible protein (IP30), interleukin (IL), latent TB infection (LTBI), mitogen activated protein kinase kinase (MAP2K), macrophage inflammatory protein (MIP), monocyte chemotactic protein (MCP), previous active TB disease (PTBD; after successful treatment), QuantiFERON Gold In-tube (QFT-G), Ras-related nuclear protein (RAN), tuberculosis (TB)

^b^ Acid-fast bacilli [AFB] sputum smear microscopy

^c^ Individuals with a positive QFT-G and negative AFB diagnostic

^d^ Individuals with previous ATBD who recently received anti-TB treatment for pulmonary ATBD (2-371 days prior to study enrollment), and post-treatment sputum conversion (based on AFB)

^e^ Controls were considered individuals with negative QFT-G and AFB diagnostic result
